# The expression and clinical significance of miR-30b-3p and miR-125b-1-3p in patients with periodontitis

**DOI:** 10.1186/s12903-022-02360-6

**Published:** 2022-08-05

**Authors:** Jinjuan Zhu, Zhihong Zhong

**Affiliations:** Department of Stomatology, China Resources and WISCO General Hospital, No. 209, Yejin Avenue, Qingshan District, Wuhan, 430080 Hubei China

**Keywords:** Periodontitis, miR-30b-3p, miR-125b-1-3p, Gingival crevicular fluid, Probing pocket depth, Clinical attachment loss

## Abstract

**Objective:**

Periodontitis is a chronic inflammatory infectious disease caused by the deposition of dental plaque on the tooth surface, leading to adverse systemic consequences. Accumulating evidence shows that dysregulated microRNAs (miRNAs) are associated with the disease severity of periodontitis. Herein, we report two novel miRNAs, miR-30b-3p and miR-125b-1-3p, in the context of periodontitis and their relationships with disease severity of periodontitis.

**Methods:**

The miRNA profiles of gingival crevicular fluid (GCF) samples were used to screen differentially expressed miRNAs (DEmiRNAs) between periodontitis patients and periodontally healthy individuals. Clinical human GCF samples were collected from 80 patients diagnosed with periodontitis (PD +) for the first time and 100 periodontally healthy individuals (PD-). The severity of periodontitis was categorized into mild/moderate (MPD) and severe (SPD) groups. The expressions of miR-30b-3p and miR-125b-1-3p were determined by quantitative real-time PCR. The levels of IL-1β, IL-6, and TNF-α were determined by ELISA methods.

**Results:**

We applied GEO2R bioinformatics tool to analyze the raw data of the GSE89081 dataset and identified miR-30b-3p (|logFC|= 1.987) and miR-125b-1-3p (|logFC|= 1.878) between periodontitis patients and periodontally healthy individuals. It was found that PPD, CAL, BOP, and the relative expression levels of miR-30b-3p and miR-125b-1-3p were all higher in the PD + group than the PD- group, in the SPD group than the MPD group (*P* < 0.05). The periodontitis patients with high-miR-30b-3p expression exhibited increased PPD, CAL, and BOP compared to those low-miR-30b-3p expression, while high-miR-125b-1-3p expression group showed significant differences on PPD and BOP from low-miR-125b-1-3p expression group (*P* < 0.05). Pearson correlation analysis demonstrated a significantly positive correlation between the levels of inflammatory cytokines, miR-30b-3p expression, and miR-125b-1-3p expression (*P* < 0.001). Results of ROC curves showed AUC of 0.878 and 0.927, sensitivity of 0.843 and 0.855, and specificity of 0.791 and 0.801, respectively, when miR-30b-3p and miR-125b-1-3p expression levels were used to diagnose periodontitis.

**Conclusion:**

These data unveiled that miR-30b-3p and miR-125b-1-3p expressions may be associated with the pathogenesis of periodontitis.

## Introduction

Periodontitis is the sixth most common human disease, with a total incidence rate of 45–50% and 11.2% of global population suffers from severe periodontitis [1]. Periodontitis is characterized by progressive destruction of tooth-supporting structure, which is generally the pathological loss of periodontal ligament and alveolar bone [2]. Periodontitis is mainly divided into necrotizing periodontal diseases, endodontic-periodontal lesions, and periodontal abscesses depending on the severity of disease at presentation [3]. Gram negative bacteria in subgingival biofilm and its products are the main cause of periodontitis [4]. The complex dynamic interactions between active herpesviruses, specific bacterial pathogens and destructive immune responses are involved in the occurrence of periodontitis [5]. Although periodontitis is normally considered a silent disease accompanied by swelling, bleeding and/or tooth mobility in the absence of pain, as an aspect of oral health, it has been proved to negatively impact patient’s physical and mental health care [6, 7]. Furthermore, existing epidemiological evidence for significant associations between periodontitis and other diseases has been confirmed in cardiovascular diseases [8], adverse pregnancy outcomes [9], respiratory diseases [10], type 2 diabetes mellitus [11], rheumatoid arthritis [12], and cancer [13]. The biological rationality of these correlations mainly depends on the low-grade inflammation associated with periodontitis [14]. Clinical examination or radiological parameters are the current methods of diagnosing periodontitis, and further evaluation occurs when the severity of periodontitis increases [15].

**Fig. 1:**
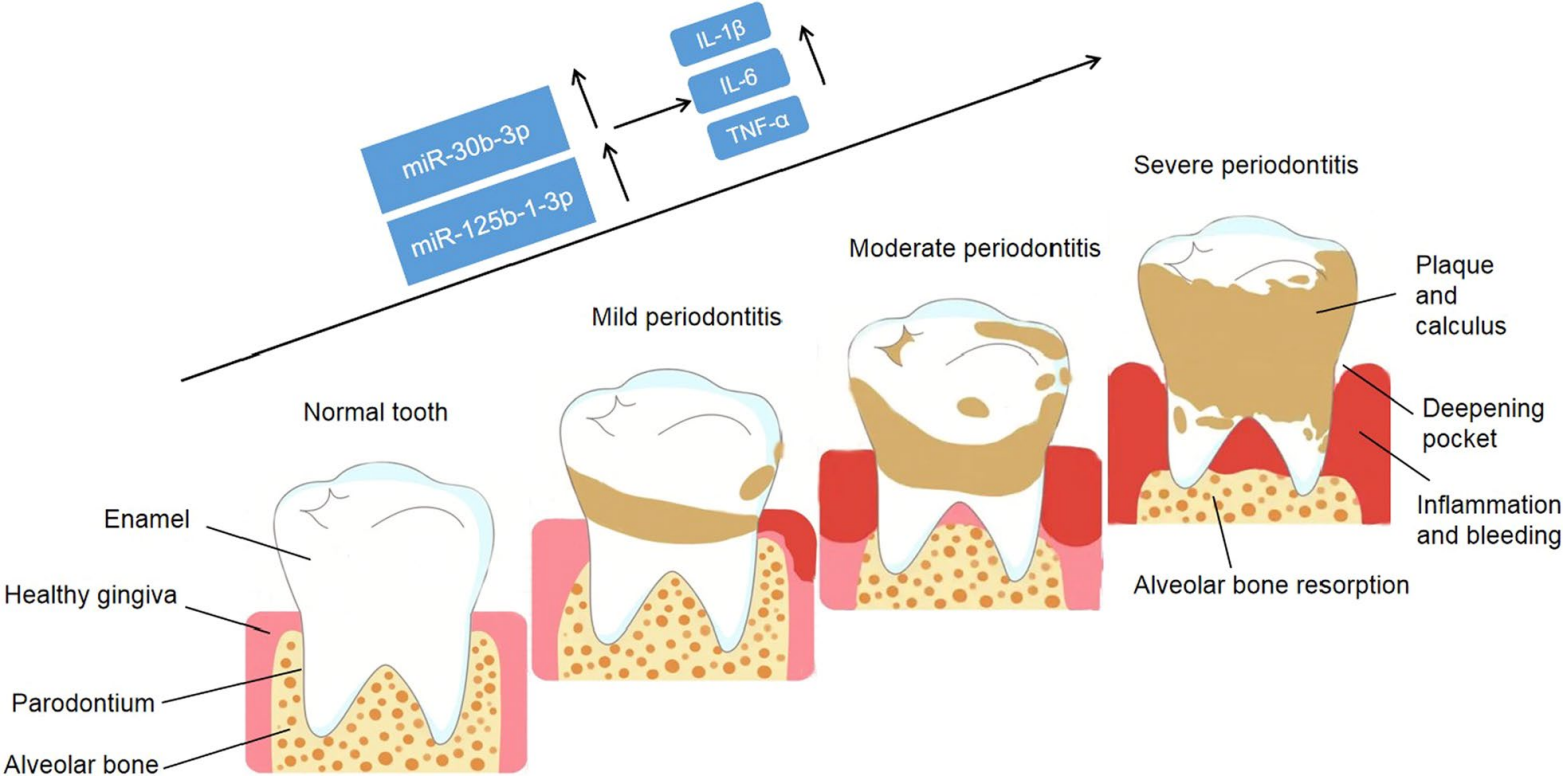
The illustrative diagram showing the anatomy of healthy versus disease gingiva and the impact of miR-30b-3p and miR-125b-1-3p as well as the released cytokines in the disease progression

Nowadays, the potential of microRNAs (miRNAs) as diagnostic and prognostic biomarkers of periodontal diseases has been evaluated [16]. For instance, a higher expression level of miR-146a were found in in patients with periodontitis than in healthy controls [17]. The clinical significance of miR-1226 in periodontitis has been studied by Du et al. [18]. They found that miR-1226 was downregulated in the gingival crevicular fluid of periodontitis patients compared with healthy volunteers and miR-1226 showed high sensitivity and specificity to discriminate periodontitis patients from healthy volunteers. Besides, miR-335-5p expression contributed to reduction in periodontal bone loss and inflammation [19]. Abnormal expression of miRNA can be used as a potential biomarker of periodontitis. The research found that lncRNA CALB2 reversed the inhibitory effect of miR-30b-3p on the differentiation of odontoblasts of human dental pulp stem cells [20], and miR-125b-1-3p was up- regulated in dental pulp stem cells [21]. Accordingly, we propose an intriguing hypothesis (Fig. 1) that increased levels of miR-30b-3p and miR-125b-1-3p in gingival dental fluid (GCF) lead to released cytokines, contributing to the disease progression of periodontitis.

## Materials and methods

### Microarray dataset analysis

The miRNA profiles of GCF samples (accessioned as GSE89081) were downloaded from the Gene Expression Omnibus (GEO, https://www.ncbi.nlm.nih.gov/gds). The GSE89081 was generated on the GPL22600 platform, including 5 healthy individuals and 3 patients with periodontitis. Differentially expressed miRNAs (DEmiRNAs) in the GCF between periodontally healthy individuals and periodontitis with log2|fold change (FC)|> 1 and adjusted *P* < 0.05 were sorted using GEO2R bioinformatics tool [22]. GEO2R software was used to identify differential gene expression, and differences were considered significant for *P* < 0.05 after Benjamini–Hochberg false discovery rate (FDR) adjustment test.

### Patients

We recruited 80 patients diagnosed with periodontitis for the first time and 100 periodontally healthy individuals from January 2020 to September 2021 at our hospital. The diagnosis of periodontitis was based on clinical and radiographic presentations in accordance with the 2018 American Academy of Periodontology (AAP) and the European Federation of Periodontology (EFP) definitions of periodontitis and staging of periodontal disease [23]: detectable periodontitis-related interdental clinical attachment loss (CAL) at ≥ 2 non-adjacent teeth or having a buccal or oral CAL of ≥ 3 mm with > 3 mm probing pocket depth (PPD) detectable at ≥ 2 teeth. The severity of periodontitis was categorized into mild/moderate and severe groups according to interdental CAL at site of greatest loss, radiographic bone loss, and tooth loss, as previously reported [24]. Patients with periodontitis were included in this study must have detailed clinical records and more than 16 remaining teeth in their oral cavities. The exclusion criteria for retrospectively analysis of patients with periodontitis were: (i) any previous periodontal treatment in the past 6 months before admission; (ii) use of antibiotics and/or anti-inflammatory drugs for 6 months; (iii) no history of systemic diseases, pneumonia, infections, surgical disease or bone metabolic disease; (iv) pregnancy or breastfeeding for women; and (v) history of SARS-CoV-2 infection. Periodontally healthy individuals should be those with PPD < 3 mm, CAL < 2 mm, bleeding on probing (BOP) < 10%, no clinical evidence of gingival inflammation, and no radiographic evidence of alveolar bone loss (ABL) [25]. Each participant signed written informed consent forms after full understanding the study aim. The study was approved by the Ethics Committee of our hospital.

### GCF sampling

The collection sites for the GCF samples in the periodontitis patients were the teeth with a CAL ≥ 6 mm. The collection sites for the GCF samples in the healthy individuals were a single-rooted tooth. On collection day, study subjects were prohibited from eating and overexercising within previous at least 2 h, eating odorous foods within previous 48 h, using usual oral hygiene practices, mouth rinses, breath fresheners, and chewing gum within previous at least 2 h, or smoking within previous at least 5 h before their assessment. Before sampling, the supragingival plaque and saliva around teeth were removed using a cotton pellet, and the teeth were air-dried and isolated with cotton rolls. The sterile Periopaper strips (Oraflow, Inc., NY, USA) were gently inserted into the periodontal pocket until mild resistance for 30 s to collect GCF samples as reported previously [26]. The strips contaminated with blood were discarded. Multiple strips were used for collecting approximately 4.8 μL of GCF for each subject. Considering the volume of GCF fully absorbed by a single strip corresponding to 1.2 μL, the sample volume was estimated according to the absorption levels of collected strips. In order to obtain a sufficient amount of GCF, multiple paper strips collected from several tooth sites were needed. The day of the analysis four pooled paper strips containing GCF of each subject were eluted in 160 μL phosphate buffered saline (PBS, pH7.2, Sigma Aldrich, USA) with a protease inhibitor cocktail. Then, the samples were vortexed and incubated on ice for 30 min following by centrifugation (12,000 × g) for 5 min at 4 °C. All eluted samples were collected into a new tube and stored at − 80 °C. Aliquots of 80 μl of each GCF sample were used to run in parallel 2 assays, one for the cytokine detection and one for the miRNA expression detection.

### Analysis of miRNA expression by quantitative real-time PCR (qRT-PCR)

Total RNA was extracted from the 80 μl GCF sample using the TRIzol reagent (Invitrogen, Carlsbad, CA, USA) following the manufacturer’s manual. The purity and concentration of RNA (OD260/280 between 1.7 and 2.1) were detected by an ultraviolet spectrophotometer. The generation of complementary DNA (cDNA) template was carried out using the TaqMan miRNA Reverse Transcription kit (Invitrogen) following the manufacturer’s manual. The expression of miR-30b-3p and miR-125b-1-3p was determined on the 7300 Real-Time PCR System (Applied Biosystems; Thermo Fisher Scientific Inc., USA) using the SYBR Green qPCR Mix kit (D7260, Beyotime, Beijing, China). The primer sequence information of miR-30b-3p was 5’-CGGCGGTGTAAACATCCTACAC-3’ (forward) and 5’-ATCCAGTGCAGGGTCCGAGG-3’ (reverse), that of miR-125b-1-3p was 5’-ACGGGTTAGGCTCTTGGGAGCT-3’ (forward) and 5’-CAGTGCGTGTCGTGGAGT-3’ (reverse), that of U6 was 5’-CTCGCTTCGGCAGCACATATACT-3’ (forward) and 5’-ACGCTTCACGAATTTGCGTGTC-3’ (reverse). The melting curve was prepared for all the reactions to confirm the precision of each sample. The cycle threshold (Ct) values were normalized to the level of U6 and results were then converted into fold change using the 2^−ΔΔCt^ formula.

### Enzyme-linked immunosorbent assay (ELISA)

The levels of IL-1β, IL-6, TNF-α, and MMP-8 in the 80 μl GCF sample was determined by ELISA methods. All ELISA procedures were performed in accordance with the protocol supplied by the kits’ manufactures (R&D Systems, USA). The GCF IL-1β, IL-6, and TNF-α concentrations were equated to a standard calibration curve.

### Statistical analysis

Statistical analysis was performed Graphpad Prism 8 (GraphPad Software, CA, USA) and SPSS version 22.0 statistical package (IBM Corp, Armonk, NY, USA) for Windows. Measurement variables are shown as mean ± standard deviation and analyzed by independent student’s t test. Categorical data were shown by number with percentage and analyzed by chi-square test or Fisher’s exact test. Pearson correlation test were used to assess the association between miR-30b-3p, miR-125b-1-3p, IL-1β, IL-6, and TNF-α levels. The receiver operating characteristic (ROC) and logistic regression analysis were performed to estimate the diagnostic values of miR-30b-3p and miR-125b-1-3p in periodontitis. The level of p < 0.05 reflects the presence of significant difference.

## Results

### Identification of GCF DEmiRNAs between periodontally healthy individuals and periodontitis

**Fig. 2:**
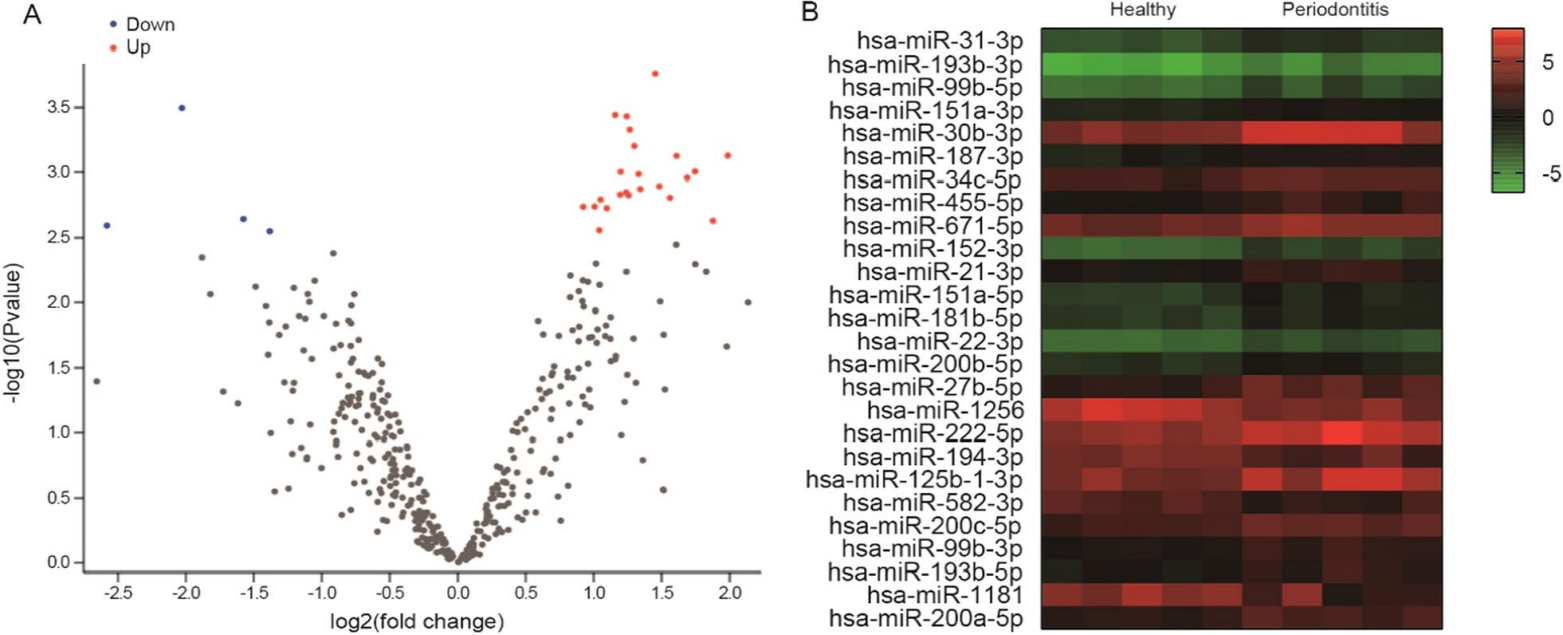
Identification of DEmiRNAs in the GCF samples between periodontally healthy individuals (n = 5) and periodontitis (n = 5) deposited in the GSE89081 dataset. There were 22 upregulated DEmiRNAs and 4 downregulated DEmiRNAs, as shown in the volcano plot (**A**) and the heatmap (**B**)

We used GEO2R bioinformatics tool to analyze the raw data of the GSE89081 dataset and identified 22 upregulated DEmiRNAs and 4 downregulated DEmiRNAs in the GCF between periodontally healthy individuals and periodontitis all which fulfilled the criteria of |log2(FC)|> 1 and corrected p < 0.05, as shown by the volcano plot (Fig. 2A). All 27 DEmiRNAs are shown by the heatmap in Fig. 2B. Among 22 upregulated DEmiRNAs, miR-30b-3p and miR-125b-1-3p ranked top 1 (|logFC|= 1.987) and top 2 (|logFC|= 1.878) were selected for further clinical validation.

### Demographics and clinical characteristics

Demographics and clinical characteristics of study subjects grouped by periodontitis (PD +) and periodontally healthy individuals (PD-), mild/moderate periodontitis (MPD) and severe periodontitis (SPD) are listed in Table 1. According to the severity of periodontitis, there were 44 periodontitis patients in the MPD group and 36 patients in the SPD group. No significant difference was found in age, gender distribution, current smoking, and the proportion of subjects with SARS-CoV-2 vaccination between PD + and PD- groups, MPD and SPD groups (*P* > 0.05). It was found that PPD, CAL, and BOP were all higher in the PD + group than the PD- group, in the SPD group than the MPD group (*P* < 0.05).

**Table 1:** Demographics and clinical characteristics

Variable	PD + (n = 80)	PD- (n = 100)	*P*	MPD (n = 44)	SPD (n = 36)	*P*
Gender/male (%)	48 (60.00%)	63 (63.00%)	0.758	26 (61.36%)	22 (58.33%)	0.823
Age (year)	40.53 ± 8.19	38.86 ± 7.77	0.164	40.61 ± 8.58	40.42 ± 7.82	0.917
Current smokers (%)	38 (47.50%)	40 (40.00%)	0.364	18 (40.91%)	20 (55.56%)	0.261
SARS-CoV-2 vaccination	18 (22.50%)	27 (27.00%)	0.604	11 (25.00%)	7 (19.44%)	0.601
PPD (mm)	3.02 ± 0.69	1.95 ± 0.45	< 0.001	2.50 ± 0.37	3.65 ± 0.41	< 0.001
CAL (mm)	3.60 ± 1.55	0.71 ± 0.50	< 0.001	2.32 ± 0.50	5.18 ± 0.67	< 0.001
BOP (%)	48.10 ± 8.13	5.73 ± 0.04	< 0.001	43.15 ± 4.95	54.15 ± 7.13	< 0.001

### Upregulation of miR-30b-3p and miR-125b-1-3p in periodontitis patients

**Fig. 3:**
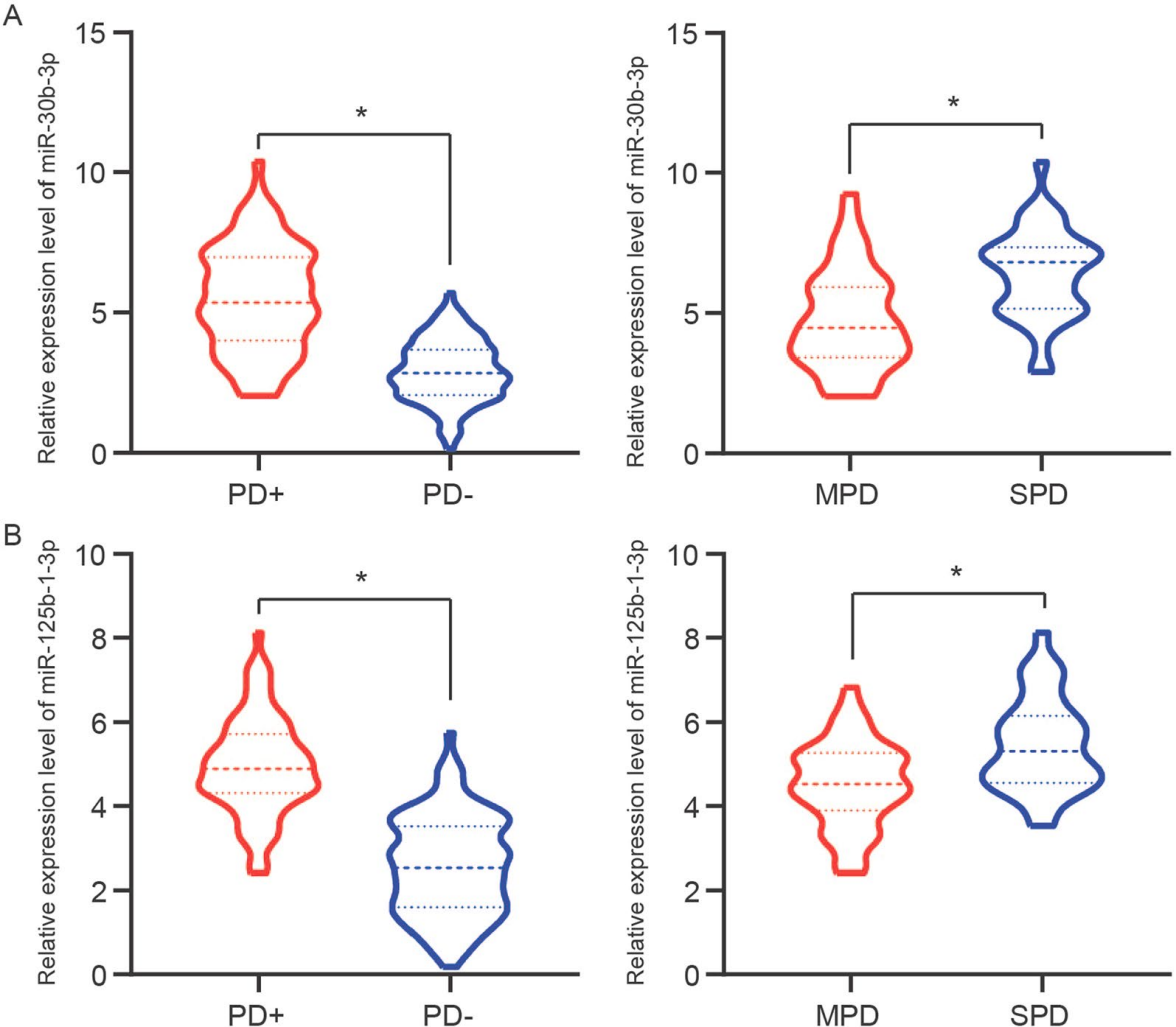
The relative expression levels of miR-30b-3p (**A**) and miR-125b-1-3p (**B**) in the GCF samples of PD + (n = 80) and PD- (n = 100) groups, in the SPD (n = 44) and MPD (n = 36) groups were determined by quantitative real-time PCR. * *P* < 0.001

The relative expression levels of miR-30b-3p and miR-125b-1-3p in the GCF samples between periodontitis patients and periodontally healthy individuals were detected by qRT-PCR. It was found that the relative expression levels of miR-30b-3p and miR-125b-1-3p were elevated in the GCF samples obtained from PD + group compared to those from PD- group (Fig. 3A, B, *P* < 0.001). Of note, the SPD group exhibited significantly higher expression levels of miR-30b-3p and miR-125b-1-3p in the GCF samples than the MPD group. We split these 80 patients with periodontitis into a high miR-30b-3p expression group (miR-30b-3p expression level ≥ 5.51, the average expression of miR-30b-3p in collected samples of periodontitis patients, n = 38) and a low miR-30b-3p expression group (miR-30b-3p expression level < 5.51, n = 42). It was showed a significant association between miR-30b-3p expression, PPD, CAL, and BOP in patients with periodontitis (Table 2, *P* < 0.05). Additionally, we grouped these 80 periodontitis patients into a high miR-125b-1-3p expression group (miR-125b-1-3p expression level ≥ 4.95, n = 39) and a low miR-125b-1-3p expression group (miR-125b-1-3p expression level < 4.95, n = 41). It was revealed that the miR-125b-1-3p expression level was associated with PPD and BOP (Table 2, *P* < 0.05) rather than CAL (*P* = 0.149). These data unveiled the high expressions of miR-30b-3p and miR-125b-1-3p were associated with the development of periodontitis.

**Table 2:** Associations between miR-30b-3p, miR-125b-1-3p expressions, the PPD, CAL, and BOP in patients with periodontitis

Variable	H-miR-30b-3p	L-miR-30b-3p	*P*	H-miR-125b-1-3p	L-miR-125b-1-3p	*P*
PPD (mm)	3.32 ± 0.58	2.75 ± 0.68	0.001	3.31 ± 0.62	2.74 ± 0.64	< 0.001
CAL (mm)	4.08 ± 1.60	3.17 ± 1.37	0.008	3.86 ± 1.62	3.36 ± 1.45	0.149
BOP (%)	51.22 ± 5.89	45.27 ± 8.88	< 0.001	50.55 ± 8.29	45.77 ± 7.35	0.008

H, High; L, low

### The levels of proinflammatory cytokines in the GCF and their association with miR-30b-3p and miR-125b-1-3p

**Fig. 4:**
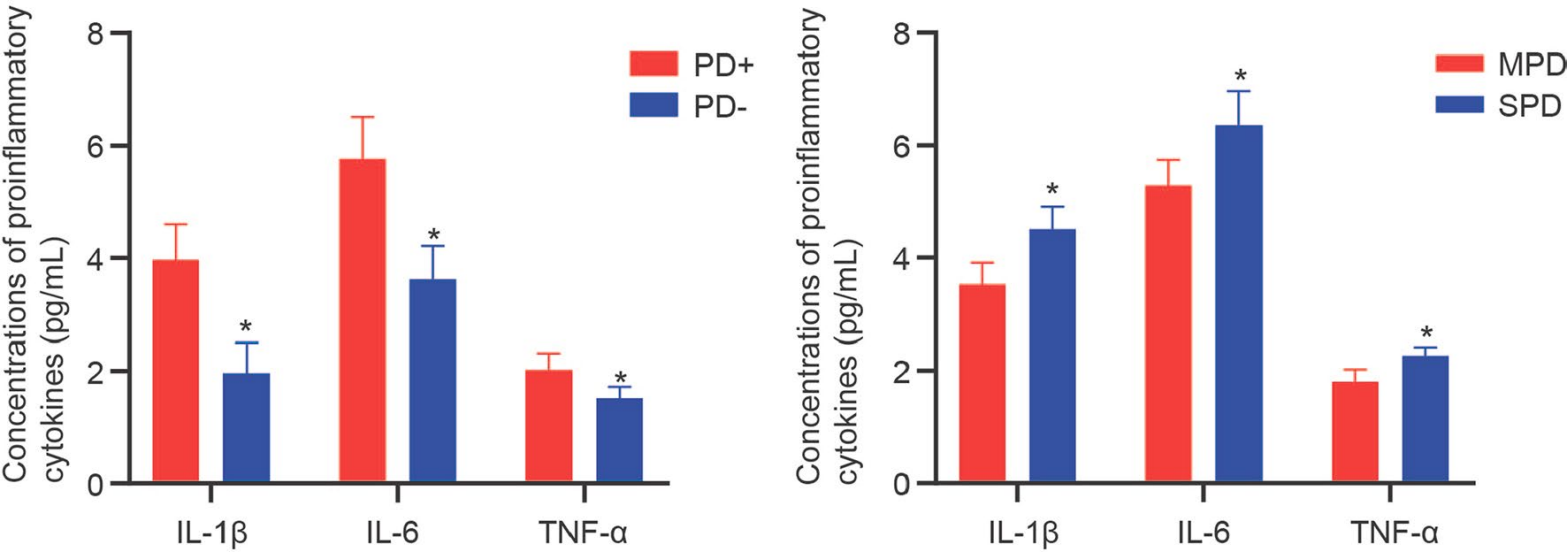
The levels of IL-1β, IL-6, and TNF-α in the GCF samples of PD + (n = 80) and PD- (n = 100) groups, in the SPD (n = 44) and MPD (n = 36) groups were determined by ELISA methods. * *P* < 0.05 compared with PD- or MPD group

**Fig. 5:**
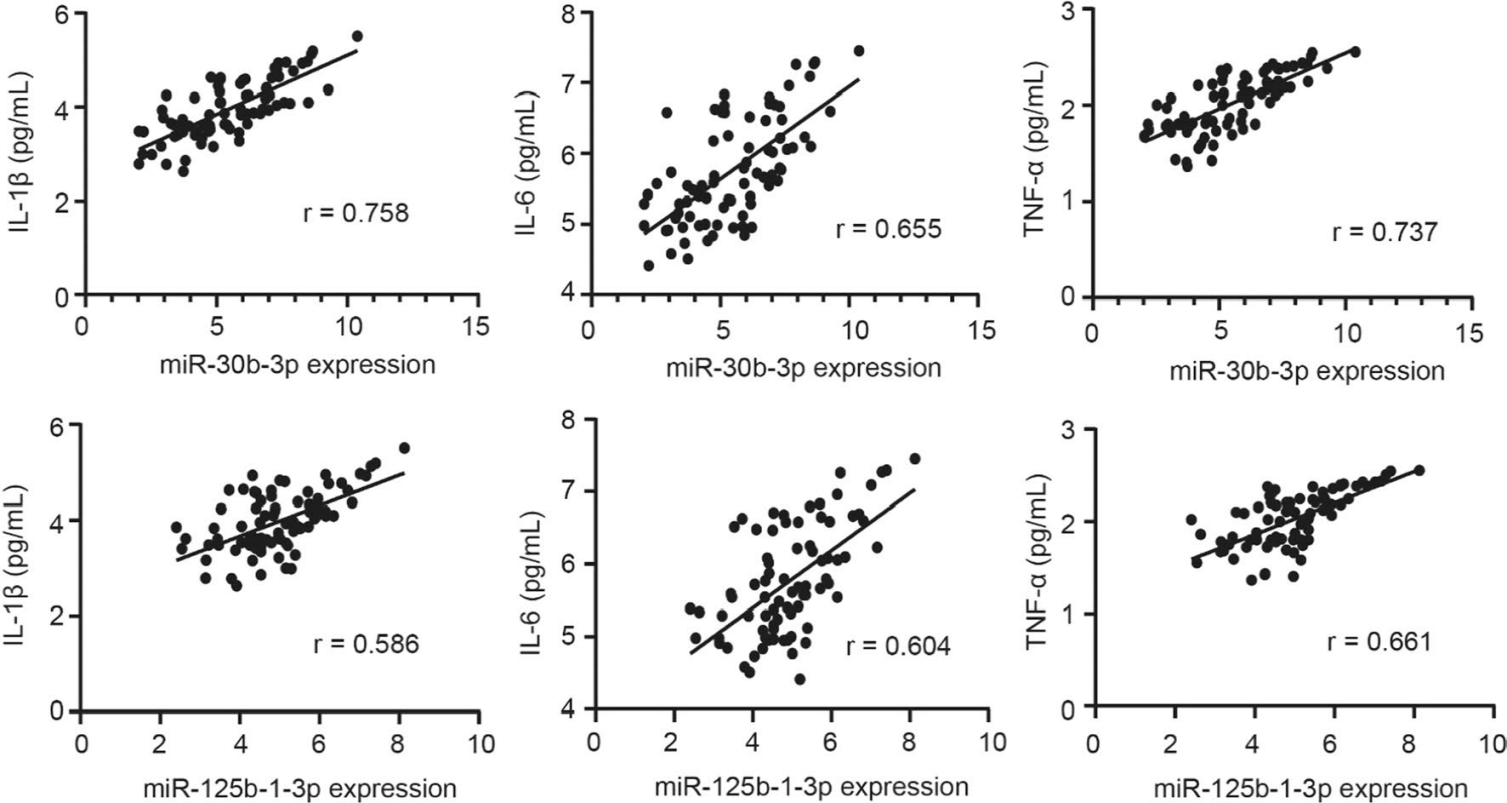
Pearson correlation analyses of the levels of inflammatory cytokines, miR-30b-3p expression, and miR-125b-1-3p

Periodontitis is a chronic inflammatory disease triggered by the host immune response. We next determined the concentrations of proinflammatory cytokines, IL-1β, IL-6, and TNF-α, in the GCF samples and their association with miR-30b-3p and miR-125b-1-3p. Results of ELISA found that the concentrations of IL-1β, IL-6, and TNF-α in the GCF samples obtained from PD + group and SPD group were elevated compared to those from PD- group and MPD group, respectively (Fig. 4, *P* < 0.05). Besides, Pearson correlation analysis demonstrated a significantly positive correlation between the levels of inflammatory cytokines and miR-30b-3p expression (*P* < 0.001), between the levels of inflammatory cytokines and miR-125b-1-3p (*P* < 0.001, Fig. 5).

### The diagnostic value of miR-30b-3p and miR-125b-1-3p in periodontitis patients

**Fig. 6:**
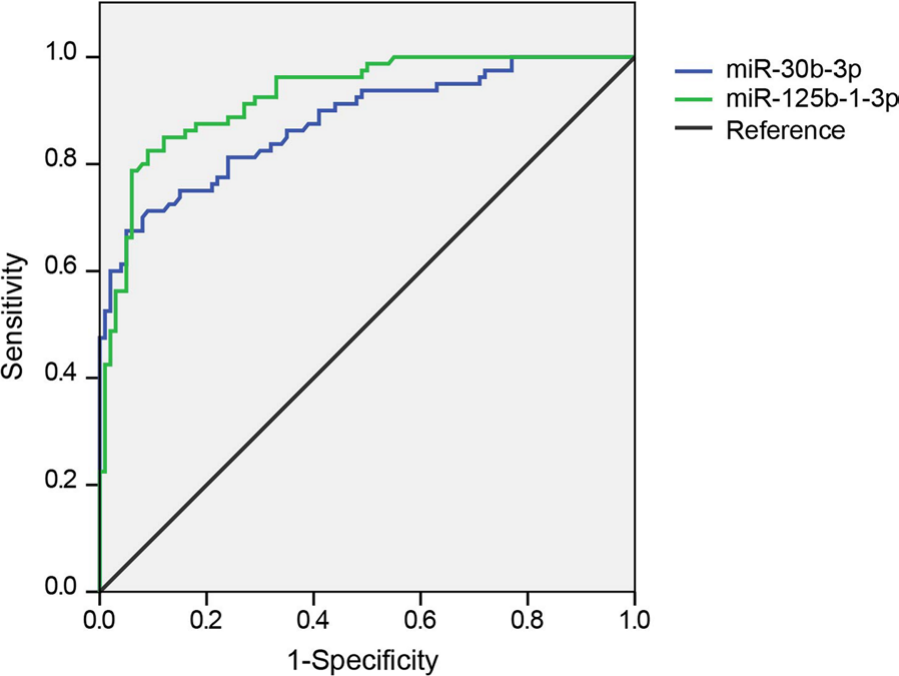
ROC curves showing relative expression levels of miR-30b-3p and miR-125b-1-3p used to differentiate between periodontitis patients and periodontally healthy individuals

Results of ROC curves showed the diagnostic values of the expression levels of miR-30b-3p and miR-125b-1-3p between periodontitis patients and periodontally healthy individuals with an AUC of 0.878 (95%CI: 0.827–0.929) and 0.927 (95%CI: 0.890–0.963), sensitivity of 0.843 and 0.855, and specificity of 0.791 and 0.801 (Fig. 6), respectively.

### Putative target genes of miR-30b-3p and miR-125b-1-3p

**Fig. 7:**
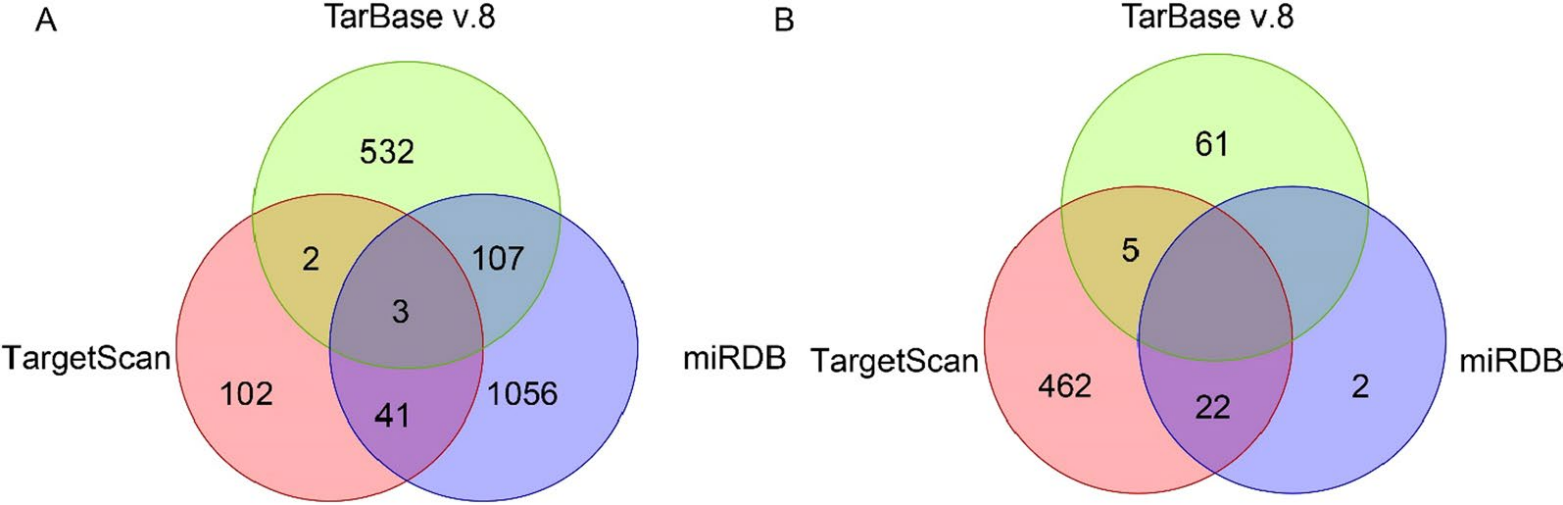
Putative target genes of miR-30b-3p (**A**) and miR-125b-1-3p (**B**) among three public miRNA-mRNA target databases

For further investigations of mechanism behind miR-30b-3p and miR-125b-1-3p in periodontitis progression, we mapped miR-30b-3p and miR-125b-1-3p into three public miRNA-mRNA target databases, TargetScan (https://www.targetscan.org/vert_80/), TarBase v.8 (https://dianalab.e-ce.uth.gr/home), and miRDB (http://mirdb.org/mirdb/index.html), to obtain target genes of two of them, respectively. There were 3 common mRNAs, AVL9 (AVL9 cell migration associated), DUSP4 (dual specificity phosphatase 4), and PXDN (peroxidasin), targeted by miR-30b-3p among the three databases (Fig. 7A). There was no common mRNA targeted by miR-125b-1-3p among the three databases (Fig. 7B), including 5 common mRNAs between TargetScan and TarBase v.8, 2 common mRNAs between TargetScan and miRDB.

## Discussion

Clinical examination or radiological parameters are commonly used in the diagnosis of periodontitis, but they are not always available in the early stage of periodontitis. Methods of predicting the development of periodontitis is of great significance to clinical management. MiRNA is considered as a novel and promising biomarker for diagnosis and development prediction of periodontal diseases as a result of developments in molecular biology.

Previous studies reported the roles of miRNAs in periodontitis. For example, miR-146a is involved in progression of periodontitis and associated with disease severity. Additionally, it is negatively correlated with the levels of pro-inflammatory cytokines [27]. Reduced expression of miR-1226 was revealed in GCF of patients with moderate or severe chronic periodontitis compared with healthy controls [28]. In our study, firstly, we identified miRNAs that expressed differentially in the GCF between periodontally healthy individuals and periodontitis through public dataset, and miR-30b-3p and miR-125b-1-3p were selected for further clinical investigations. MiR-30b-3p was identified as a new biomarker of cancers such as prostate cancer [29], ovarian cancer [30], and gastric cancer [31]. MiR-125b-1-3p was related to various diseases including severe asthma [32], atherosclerosis [33], and hypercholesterolemia [34]. The present study explored the expression patterns and clinical significance of miR-30b-3p and miR-125b-1-3p in periodontitis. The qRT-PCR analysis showed that elevated miR-30b-3p and miR-125b-1-3p of GCF were found in PD + group when it compared with PD- group, and this uptrend was more obviously in SPD group than MPD group.

In order to confirm the correlation between these two miRNAs and clinical characteristics, we assigned periodontitis patients to high miRNA expression group and low miRNA expression group. Gingivitis is well recognized in clinical and is a potential manifestation of periodontitis. A site of gingivitis with consistent bleeding can lead to a high risk of attachment loss [35]. PPD, CAL, and BOP are vital clinical parameters for periodontitis [36]. There was a close correlation among PPD, CAL, and BOP. The probability of BOP is significantly higher when PPD is greater than or equal to 5 mm. High incidence of BOP leads to the occurrence of CAL [37]. We observed the miR-30b-3p expression level was significantly related with PPD, CAL, and BOP, while miR-125b-1-3p only shows significance with regard to PPD and BOP, but not CAL. PPD and CAL are parameter indirectly associated with bone resorption. The results may suggest upregulation of miR-30b-3p may have a closer association with alveolar bone resorption than miR-125b-1-3p in the context of PD, which should be investigated in future functional studies based on dental pulp stem cells. These findings were similar to a previous research to some extent, indicating patients with periodontitis showed lower level of miR-1226 in GCF than healthy controls, and a negative correlation was found between miR-1226 expression, PPD, CAL, and BOP [18]. Nonsurgical periodontal treatments, including quadrant-wise debridement, scaling and root planning, full-mouth scaling, full-mouth disinfection (FMD), FMD with adjuvant erythritol air-polishing, and drug therapies were applied in periodontitis patients, with the purpose to improve both clinical and microbiologic outcomes [38, 39]. With regard to the therapeutic implications exerted by clinical significance of miR-30b-3p and miR-125b-1-3p in periodontitis, as previously reported [40], tetramethylpyrazine is a promising agent and its anti‑inflammatory and anti‑apoptotic effects achieved by targeting miR‑302b confer a protection to periodontal ligament stem cells from injuries induced by periodontitis. Developing agents targeting miR-30b-3p and miR-125b-1-3p thus reducing inflammation may be a new modality for nonsurgical periodontal treatments.

In periodontitis, the existence of inflammation is consistent with the severity of the disease. The disease progression is determined by the complex interaction driven by the regulation of immune inflammatory host response [41]. Subgingival bacteria and their products induce inflammatory cells, which destroy periodontal tissue and maintain an inflammatory response. The presence of inflammatory cytokines is involved in immune response in destructive periodontitis [42]. IL-6 and TNF-α level were regulated by miR-21 in macrophages of periodontitis. IL-6 and TNF-α were decreased as a result of overexpression of miR-21, and downregulated miR-21 leaded to increased IL-6 and TNF-α [43]. Our study examined the concentrations of proinflammatory cytokines and investigated their correlation between miR-30b-3p and miR-125b-1-3p. ELISA results manifested that PD + group and SPD group had higher level of IL-1β, IL-6, and TNF-α in the GCF samples than PD- group and MPD group, respectively. It was observed that these proinflammatory cytokines levels were significantly positively correlated with miR-30b-3p and miR-125b-1-3p expression. Afsar et al. found that miR-30b was associated with cytokine production in myeloid inflammatory cells [44]. Likewise, miR-30b-3p was found as one of upregulated miRNAs in children with severe pneumonia, implying its close association with inflammatory diseases [45]. Of course, miR-125b-1-3p has been also previously reported for its pro-inflammatory effects regulated by NF-κB in diverse cell types under various stimuli [46]. Increased expression of miRNAs such as miR-23a and miR-146a leads to deterioration of periodontitis. MiR-23a and miR-146a yield AUC of 0.857 and 0.886, respectively, for diagnosing periodontitis [47]. Our study used miR-30b-3p and miR-125b-1-3p expression levels to diagnose periodontitis by ROC analysis and observed that miR-30b-3p and miR-125b-1-3p yield AUC of 0.878 and 0.927, respectively.

## Conclusion

All in all, the data in this study demonstrated overexpression of miR-30b-3p and miR-125b-1-3p were associated with the development and progression of periodontitis, both of which showed diagnostic values for periodontitis. RNA-sequencing technology to profile miRNAs by ourselves rather than analyzing historical data only in the GCF between periodontitis and periodontally healthy individuals is warranted in further study to validate whether miR-30b-3p and miR-125b-1-3p are unique or common in the context of periodontitis. In addition to that, mechanism of miR-30b-3p and miR-125b-1-3p action on bone resorption or inflammation reaction in periodontitis by targeting related mRNAs should be explored by functional assays under in vitro and in vivo setting.

## Data Availability

The dataset (accession number: GSE89081) analyzed during the current study is available in the Gene Expression Omnibus (https://www.ncbi.nlm.nih.gov/geo/query/acc.cgi?acc=GSE89081).

## Abbreviations

miRNAs: MicroRNAs
GCF: Gingival crevicular fluid
DEmiRNAs: Differentially expressed miRNAs
PD-: Periodontally healthy individuals
PD +: Periodontitis
MPD: Mild/moderate periodontitis
SPD: Severe periodontitis
AAP: American academy of periodontology
EFP: European federation of periodontology
CAL: Clinical attachment loss
PPD: Probing pocket depth
BOP: Bleeding on probing
ABL: Alveolar bone loss
PBS: Phosphate buffered saline
qRT-PCR: Quantitative real-time PCR
cDNA: Complementary DNA
ROC: Operating characteristic
Ct: Cycle threshold
